# Scutellarin is Highly Likely to be Responsible for Drug-Drug Interactions Mediated by Hepatic Organic Anion-Transporting Polypeptide1B3

**DOI:** 10.1007/s11095-020-02950-5

**Published:** 2020-10-29

**Authors:** Jianming Liu, Yongmei Guo, Yanqi Xu, Li Yuan, Huiting Zhu

**Affiliations:** 1 School of Pharmacy, Nanchang University, Nanchang, Jiangxi 330031 People’s Republic of China; 2 School of Pharmacy, Jiangxi Medical College, Shangrao, Jiangxi 334000 People’s Republic of China; 3 Department of Pharmacy, Ji’an Maternal and Child Health Hospital, Ji’an, Jiangxi 343000 People’s Republic of China; 4 Pharmacy Department, The Affiliated Children’s Hospital of Nanchang University, Nanchang, Jiangxi 330006 People’s Republic of China; 5 Pharmacy Department, Jiangxi Provincial Children’s Hospital, Nanchang, Jiangxi 330006 People’s Republic of China

**Keywords:** drug-drug interactions (DDI), organic anion transporting polypeptide 1B3 (OATP1B3), rosuvastatin, scutellarin

## Abstract

**Purpose:**

Scutellarin, a flavonoid derived from the plant Erigeron breviscapus, is currently widely used to treat cerebrovascular diseases, liver-related diseases, and hyperlipidemia in china and other East Asian countries. This study was to investigate the effect of scutellarin on the uptake of rosuvastatin in HEK293T cells expressing human organic anion transporting polypeptide 1B3 (hOATP1B3) and rat OATP1B2 (rOATP1B2), respectively, and the effect of scutellarin on the pharmacokinetics of rosuvastatin in rats.

**Methods:**

The newly established HEK293T cells expressing hOATP1B3 and rOATP1B2 were used to examine the effects of scutellarin and positive controls on *in vitro* rosuvastatin transport. After co-feeding with scutellarin, the rosuvastatin area under the plasma concentration-time curve (AUC_0–24h_), the peak plasma drug concentration (*C*_max_), elimination half-life (*t*_1/2_), time to reach *C*_max_ (*t*_max_), clearance (CL) and apparent clearance (CL/F) of rosuvastatin were determined in rats.

**Results:**

Scutellarin inhibited hOATP1B3- and rOATP1B2-mediated rosuvastatin uptake (IC50: 45.54 ± 6.67 μM and 27.58 ± 3.97 μM) *in vitro* in a concentration-dependent manner. After co-feeding with scutellarin, the AUC_0–24h_ and *C*_max_ of rosuvastatin in rats increased to 27.4% and 37.7%, respectively. The *t*_1/2_ and *t*_max_ of rosuvastatin showed no significant change. Moreover, scutellarin caused 29.2% and 28.1% decrease in the CL and CL/F of rosuvastatin.

**Conclusion:**

Scutellarin may inhibit the hOATP1B3- and rOATP1B2-mediated transport of rosuvastatin *in vitro*, and exerts a moderate inhibitory effect on the pharmacokinetics of rosuvastatin in rats. Scutellarin is highly likely to participate in drug-drug interactions, as mediated by OATP1B3 in humans.

## Introduction

Traditional Chinese Medicine still plays an important role in protecting public health in countries like China and Japan (1). Scutellarin (SCU), a flavonoid derived from the plant Erigeron breviscapus, is an active ingredient of a variety of Chinese herbal medicine, with broad pharmacological effects and clinical applications (2). SCU is currently widely used to treat cerebrovascular diseases, liver-related diseases, and hyperlipidemia (3,4). It is estimated that over ten million Chinese patients are taking SCU and its related drugs annually (5). Meanwhile, drug–drug interactions (DDI) adverse events have attracted increasing attention (6). At the pharmacokinetic level, various interactions between traditional Chinese medicine and drugs may occur, especially in the liver (7). The metabolism and disposition of drugs in the liver plays a critical role in both drug detoxification and drug toxification (8). Transporters play a vital role in drug pharmacokinetics, and competing for them is crucial for drug uptake, distribution, and elimination (9,10). The organic anion-transporting polypeptides (OATPs) may mediate DDI when drugs pass through the hepatic sinusoids (11). Both genetic variation and DDI may change the transport function of human OATP1B1(hOATP1B1) and human OATP1B3(hOATP1B3) thus frequently trigger severe adverse events, for example, statin-induced rhabdomyolysis (12). In previous studies, we have shown that SCU affects the uptake of rosuvastatin (RSV) in rat primary hepatocytes and HEK293T-hOATP1B1 cells (13). Besides, hOATP1B1 and hOATP1B3 proteins share 80% identity in amino acid sequences in humans (14) and have common substrates like rifampicin (RIF) (15) and RSV (16). Rat OATP1B2(rOATP1B2) also locates in the sinusoidal plasma membrane and RSV is also its substrate (17). In rodents, OATP1B2 is considered as the closest ortholog to hOATP1B1 and hOATP1B3 (18). *In vivo* distribution of SCU is mainly in hepatocytes, the main target cells of HMG-GoA reductase statins (19). Up to now, a large number of studies have confirmed the pharmacokinetic interaction between SCU and statins (2,20). Though SCU is known to affect the uptake of RSV in the cells expressing hOATP1B1 (13), it remains unclear whether SCU affects RSV uptake in cells expressing hOATP1B3 or rOATP1B2, or is involved in pharmacokinetic DDI. Therefore, the *in vitro* or *in vivo* mechanisms underlying uptake of RSV remains to be clarified. Considering that most hOATP1B1 inhibitors will also inhibit hOATP1B3 clinically, SCU may be involved in DDI of these transporters.

In this study, we aimed to determine the potential effect of SCU on the activity of hOATP1B3 and rOATP1B2 transporters *in vitro* and to assess the effect of SCU on the pharmacokinetics of RSV in rats.

## Materials and Methods

### Materials

SCU (purity ≥98%, HPLC) was purchased from Kunming Double Star Technology Development Co.Ltd. RSV calcium (purity ≥98%, HPLC) was purchased from Zhejiang Xindonggang Pharmaceutical Co.Ltd. Atorvastatin (internal standard) and RIF (purity ≥98%, HPLC) were sourced from the National Institute for the Control of Pharmaceutical and Biological Products. Sodium butyrate (purity ≥98%, HPLC) was provided by Sigma-Aldrich. Glycyrrhizin acid (GA) (purity ≥98%, HPLC) was purchased from Shanghai Yuanye Biotechnology Co.Ltd. Polyclonal antibodies against hOATP1B3, rOATP1B2 and Na+/K + -ATPase were sourced from Proteintech Co. Ltd. The transporter gene plasmid was provided by Shanghai Jikai Gene Co. Ltd. Human embryonic kidney 293 T (HEK293T) cell line was sourced from the Institute of Basic Medical Sciences, Chinese Academy of Medical Sciences (Beijing, P. R. China).

### Cell Culture and Transfection

HEK293T cells were cultured at 37°C in Minimum Essential Medium supplemented with 10% FBS (Sigma-Aldrich), 100 U·mL^−1^ penicillin, and 100 mg·mL^−1^ streptomycin in a humidified 5% CO_2_ atmosphere. HOATP1B3 and rOATP1B2 cDNA were respectively transfected into HEK293T cells according to the manufacturer’s instructions. Cells were selected by adding G418 (1 mg·mL^−1^) to the culture medium to obtain HEK293T-hOATP1B3 and HEK293T-rOATP1B2 cells. Cell transfection is successful and it can be used for a transport experiment after about 3 passages. Then, the HEK293T cells selected by 6 mg·mL^−1^ blasticidin and colonies were picked up. The HEK293T cells with the highest transport activities were used for functional analysis. To induce the expression of hOATP1B3 and rOATP1B2, sodium butyrate (2.5 mM) was added to the medium 24 h before use.

### Western Blot Analysis

According to a previous study (13), the cell suspension was prepared by digesting HEK293T, HEK293T-MOCK, HEK293T-hOATP1B3, and HEK293T-rOATP1B2 cells at the logarithmic proliferative phase with trypsin. Cells were lysed with SDS protein lysate, and cell proteins were extracted by centrifugation. The total protein concentration was determined using the 2-quinolinic acid (BCA) method. Then, 10% sodium dodecylbenzene sulfonate polyacrylamide was used to transfer the proteins to polyvinylidene fluoride membrane (PVDF) by gel electrophoresis. The samples were blocked in 5% milk at room temperature for 1 h, then the primary antibody was added and incubated overnight at 4°C. The membrane was briefly rinsed for 5 min in TBST, which was repeated 3 times. The secondary antibody and goat anti-rabbit antibody conjugated to horseradish peroxidase were added and incubated at room temperature for 1 h, and then the membrane was rinsed 3 times with TBST, 10 min each time. The ODYSSEY imaging system was applied to add ECL chemiluminescence developer for image acquisition.

### Transport Assay

HEK293T-MOCK, HEK293T-hOATP1B3, and HEK293T-rOATP1B2 cells were seeded in a 24-well plate coated with poly-L-lysine at an initial density of 2 × 10^5^ cells/well. 24 h after the cell seeding plate, sodium butyrate was added to induce transporter protein expression for 24 h. Then, the cells were washed twice and pre-incubated with Krebs-Henseleit buffer at 37°C for 10 min, and then incubated in 200 μL of Krebs-Henseleit buffer cells containing RSV (50 μM) and SCU (50 μM) or the vehicle (0.2% dimethyl sulfoxide) at 37°C for 10 min (13). RIF (50 μM) was used as an inhibitor (positive control) against OATP1B3.GA (20 μM) was applied as an inhibitor against OATP1B2 (positive control) (21). RSV (50 μM) was incubated with HEK293T-hOATP1B3 and HEK293T-rOATP1B2 cells in the presence of SCU with increasing concentrations (0.01–200 μM) for 10 min. After removing the incubation buffer, the uptake reaction was terminated by adding ice-cold Krebs-Henseleit buffer, then discarded all the solutions and lysed with water by repeated freezing and thawing. Aliquots (200 μL) were vortexed and centrifuged for 5 min at 18000 g. The concentration of RSV was determined by the liquid chromatography with tandem mass spectrometric detection (LC/MS/MS) and the remaining 50 μL aliquots of the cell lysis solution were used to determine the protein concentration with BSA as a standard. The RSV uptake is expressed as a percentage of the control.

### LC/MS/MS Assay for RSV

The RSV concentrations accumulated in cells were determined using a validated LC/MS/MS method (13). The samples were prepared by adding 100 μL of internal standard solution (200 ng·mL^−1^, atorvastatin diluted in dimethyl sulfoxide) to 100 μL of cell lysates. Mass spectrometric detection was performed with the assistance of an API 4000+ triple quadrupole mass spectrometer (AB SCIEX, Boston, USA). Multiple-reaction monitoring was conducted to monitor the analytes and internal standards. The mass conditions are as follows: ion spray voltage (−4500 V), ion source gas 1 (N_2_, 60 Arb), ion source gas 2 (N_2_, 65 Arb), curtain gas (N_2_, 30 Arb), source gas temperature (550°C), collision gas (N_2_, 10 Pa). The selected mass transitions were m/z 480.2 → 418.1 for RSV and m/z 557.6 → 397.1 for atorvastatin, respectively.

### Pharmacokinetic Profile of RSV *In Vivo*

Male Sprague-Dawley rats aged 6–8 weeks and weighing between 230 and 260 g were obtained from the Experimental Animal Center of Nanchang University. All of the rats were allowed to adapt the environment in the breeding room with ideal laboratory conditions (temperature of 23–25°C, the relative humidity of 45–55%, 12 h light/12 h darkness cycle) and were given free access to standard diet and water. The study was approved by the Ethical Committee of Jiangxi Medical College, and all experiments were carried out in accordance with the requirements of the Animal Care and Use Committee of Jiangxi Medical College. After experiments, the rats were euthanized by intraperitoneal injection of 3% sodium pentobarbital.

All rats were acclimatized for 5 days before the experiments. The experimental rats were randomly divided into two groups, 6 in each group: the RSV group (RSV 10 mg·kg^−1^ + 0.2% CMC-Na) and the RSV + SCU group (RSV 10 mg·kg^−1^ + SCU 50 mg·kg^−1^). For the RSV, 0.9% sodium chloride was used to prepare a suspension of 1 mg·mL^−1^; for the SCU, 0.2% CMC-Na was used to prepare a suspension of 5 mg·mL^−1^. The gavage volume of both groups was 10 mL·kg^−1^. The concentrations used in the animals were converted from the clinical dose. The treatment was administered by gavage in the morning before feeding. For rats, SCU or 0.2% CMC-Na (vehicle) was administered immediately after the RSV administration. In each group, 6 rats were maintained throughout the study, and they were used for jugular vein blood sampling (0.2 mL) at 0 (before), 0.25 0.5, 1.0, 1.5, 2.0, 4.0, 6.0, 8.0, 12.0, 18.0, 24.0 h after treatment, so as to obtain the concentration-time profile data. A heparinized 0.9% NaCl injectable solution was applied to compensate for blood loss after each blood sampling. The sampled blood was stored in a heparinized tube, and the plasma was separated by centrifugation, and then stored at −80°C for further analysis. Plasma RSV concentrations were quantified using LC/MS/MS. *C*_max_ and *t*_max_ were obtained by analyzing the concentration-time data. The area under the RSV concentration-time curve from 0 to 24 h (AUC_0–24h_) was calculated according to the linear trapezoidal rule. *k* represents the elimination rate constant as determined from the terminal slope of the log concentration-time plot and the elimination half-life (*t*_1/2_) = 0.693/*k*. The clearance as CL = Dose/AUC_0–∞_. The apparent systematic clearance (CL/F) was calculated as CL/F = Dose/AUC_0-∞_/weight. The lower limit of quantification (LLOQ) of plasma was 1 ng·mL^−1^, the dynamic range of plasma was 1–800 ng·mL^−1^, and the intra- and inter-day coefficients of variation were less than 10%.

### Statistical Analysis

*In vitro* data from three independent experiments were analyzed using one-way ANOVA, followed by a Dunnett’s post hoc test or by Student’s t test. The AUC_0–24 h_, *t*_1/2_, *C*_max_, *t*_max_, CL and CL/F values of RSV after treatment with placebo and SCU were analyzed using paired Student’s t test. Unless otherwise stated, the results are expressed as mean ± standard deviation (SD) in the text, tables, and figures. All data were analyzed using SPSS software (version 19.0, Chicago, IL). *P* < 0.05 was considered as statistically significant.

## Results

### Transporter Expression in HEK293T by Western Blotting

**Fig. 1:**
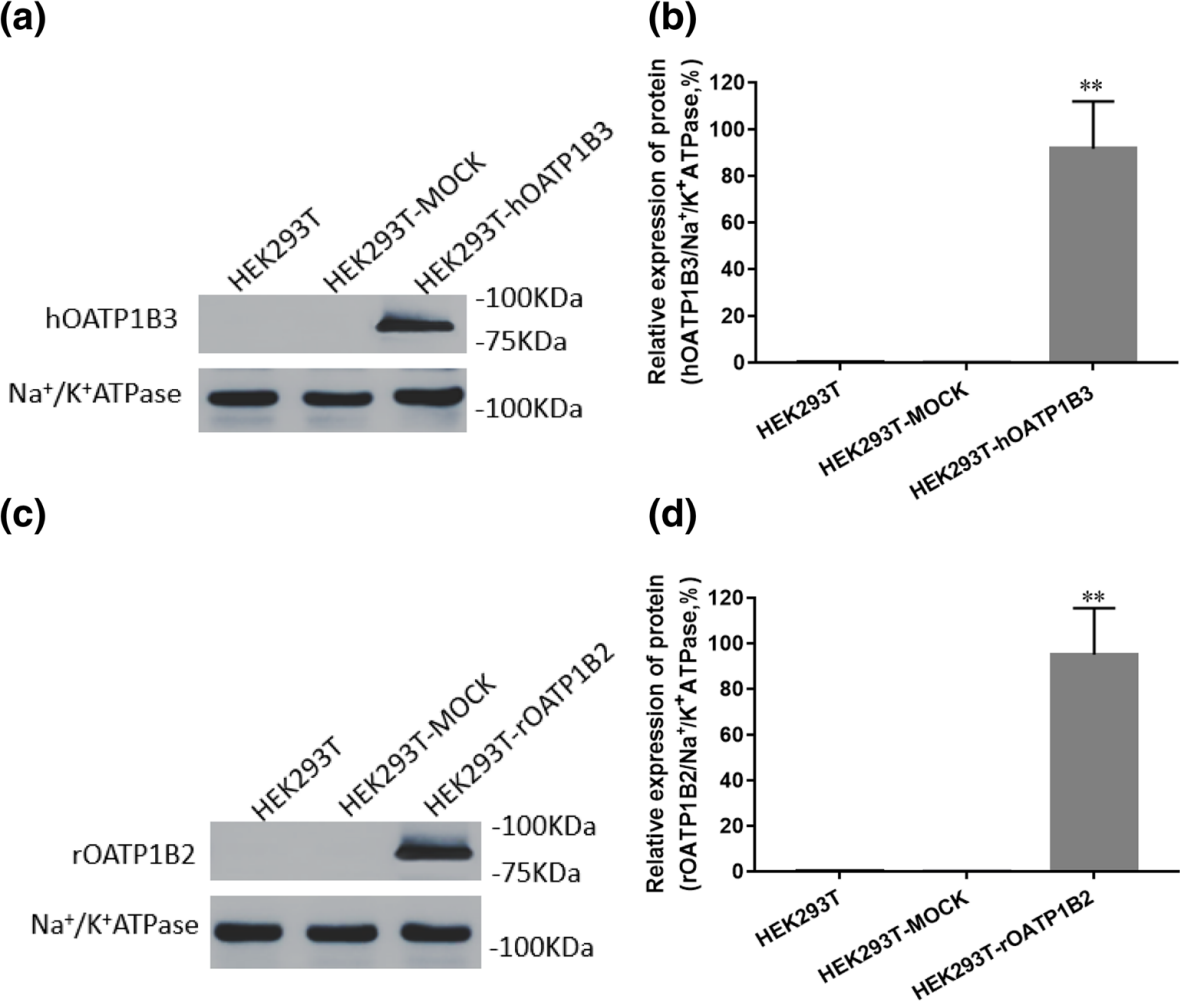
The protein expression of hOATP1B3 and rOATP1B2 in cells. The immunoblots shown in **a** & **c**. Results of integrated optical density analysis is shown in **b** & **d**. (mean ± SD, *n* = 3). Na^+^/K^+^-ATPase is a membrane protein. **indicates a significant difference compared with the HEK293T-MOCK group (*P* < 0.01).

The results showed that the blank HEK293T and HEK293T-MOCK cells contained barely any target proteins, while the western blotting of hOATP1B3 and rOATP1B2 transfected cell lines exhibited a distinctive band, indicating that HEK293T cells successfully expressed the target gene after transfection, as shown in Fig. 1.

### *In Vitro* Impact of SCU on the hOATP1B3- and rOATP1B2-Mediated Uptake of RSV

**Fig. 2:**
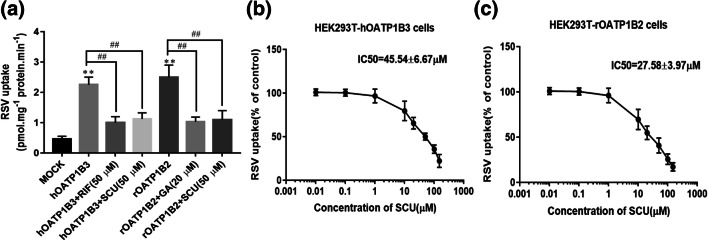
Inhibition of hOATP1B3- and rOATP1B2-mediated RSV transport. SCU (50 μM), RIF (20 μM), and GA (50 μM) inhibition on hOATP1B3 and rOatp1b2-mediated RSV in cells (**a**). SCU inhibited the hOATP1B3- (**b**) and rOATP1B2-(**c**) mediated RSV uptake (50 μM) in a concentration-dependent manner (the data are denoted as a percentage of control (RSV alone)). All data is indicated by mean ± SD (n = 3). **indicates a significant statistical difference from HEK293T-MOCK cells (*P* < 0.01), ^##^ indicates a significant statistical difference from the RSV alone (*P* < 0.01).

The RSV uptake and inhibition experiment confirmed that RSV was transported by hOATP1B3 and rOATP1B2, and the uptakes in HEK293T-hOATP1B3 and HEK293T-rOATP1B2 cells were substantially higher than in HEK293T-MOCK cells (4.9-fold and 5.7-fold). RIF (50 μM) inhibited RSV uptake in HEK293T-hOATP1B3. GA (20 μM) inhibited RSV uptake in HEK293T-rOATP1B2 cells, and SCU (50 μM) inhibited RSV uptake in HEK293T-hOATP1B3 and HEK293T- rOATP1B2. SCU exerted a notable inhibitory effect on the hOATP1B3- and rOATP1B2-mediated uptake of RSV in a concentration-dependent manner, and the half-maximal inhibitory concentration (IC50) values were 45.54 ± 6.67 μM (95% CI: 38.04, 53.04) and 27.58 ± 3.97 μM (95% CI: 23.08, 32.08), respectively, as shown in Fig. 2.

### *In Vivo* Impact of SCU on the Pharmacokinetics of RSV

**Fig. 3:**
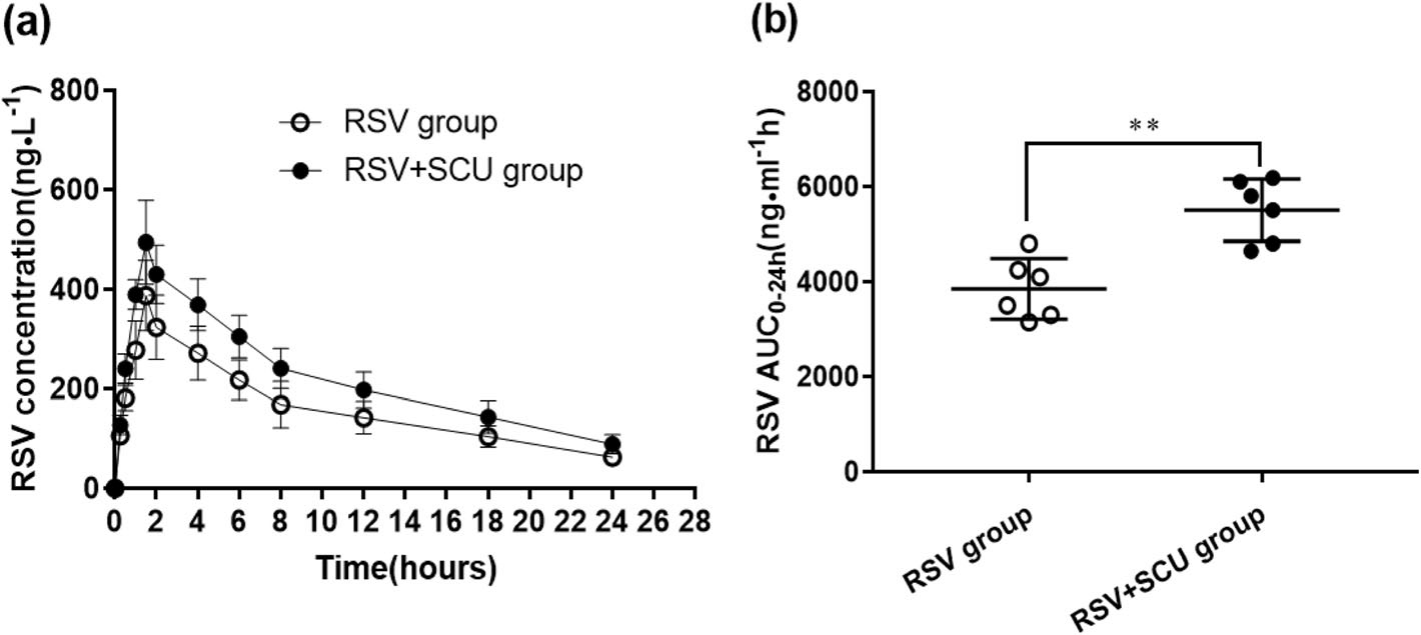
Average plasma concentration-time profiles of RSV following an oral administration of RSV (10 mg·kg^−1^) to rats in the presence and absence of SCU (50 mg·kg^−1^) (**a**) and individual values for AUC_0–24h_ (**b**) (mean ± SD, *n* = 6). **indicates a significant statistical difference from RSV group (*P* < 0.01).

Table I lists the main pharmacokinetic parameters of RSV. Figure 3 indicates the average plasma concentration profile of RSV when combined with the 0.2% CMC-Na or SCU. Treatment of SCU led to a significant increase in the *C*_max_ of RSV by 27.4% (*P* = 0.04) and AUC_0–24 h_ by 37.7% (*P* = 0.006), respectively. Subsequent to SCU treatment, the CL and CL/F of RSV was reduced significantly by 29.2% (*P* = 0.007) and 28.1% (*P* = 0.008) compared with the RSV groups, respectively. And there was no significant impact on the observed *t*_1/2_ and *t*_max_ of RSV.

**Table I:** Pharmacokinetic parameters of RSV

Parameters	RSV group	RSV+ SCU group	*P* value
Half life (*t*_1/2_, h)	4.96 ± 1.77	5.07 ± 1.95	0.593
*t*_max_ (h)	1.46 ± 0.15	1.51 ± 0.18	0.547
*C*_max_ (ng·mL^−1^)	387.67 ± 70.79	494.01 ± 84.53**	0.040
CL (L·h^−1^)	10.68 ± 1.88	7.50 ± 1.12**	0.009
CL/F (L·h^−1^·kg^−1^)	2.67 ± 0.47	1.92 ± 0.28**	0.008
AUC_0–24h (_ng·mL^−1^·h)	3841 ± 675	5287 ± 766**	0.006

The data is expressed as mean ± SD, (n = 6). *t*_max_ (time to reach *C*_max_) data is expressed as medians with range. AUC_0–24 h_, area under the plasma drug concentration-time curve from 0 to 24 h; *C*_max_, peak plasma drug concentration;*t*_1/2_, elimination half-life; CL, clearance; CL/F, apparent clearance. **indicates a significant statistical difference from the RSV group (*P* < 0.01)

## Discussion

In this study, we focused on the interactions of SCU with human hepatocellular uptake transporter hOATP1B3 and rOATP1B2. First, a single transporter cDNA was successfully transfected into the HEK293T cell line (Fig. 1). Then, we observed that HEK293T-hOATP1B3 and HEK293T-rOATP1B2 cells had higher uptake (approximately 4.9-fold and 5.7-fold) compared with HEK293T-MOCK cells. RIF (50 μM) and GA (20 μM) significantly reduced the higher uptake of RSV in transporter transfection cells. SCU significantly inhibited the hOATP1B3-, and rOATP1B2-mediated RSV uptake *in vitro* in a concentration-dependent manner, with IC50 values of 45.54 ± 6.67 μM and 27.58 ± 3.97 μM, respectively (Fig. 2). HEK293T cells are one of the commonly used *in vitro* models to study the correlation between drug transporters and substrates (21). In these HEK293T cells, the transfection rate was determined to be greater than 80%, which could meet our design requirements. In addition, according to Dong’s research method, the results of the cytotoxicity test showed that SCU (50 μM), RSV (50 μM), and GA (20 μM) posed no significant toxicity to cells, consistent with studies by Dong (21) and Liu (13). As a widely used active ingredient in Chinese medicine, we are also concerned about the concentration of SCU *in vivo*. Haisheng You *et al*. (22) found that the *C*_max_ and AUC_0-__∞_ of SCU (80 mg·kg^−1^) by oral gavage were 288.0 ± 75.2 μg·L^−1^ and 5.9 ± 1.7 μg·mL^−1^·h in rats. Zhong *et al*. (23) found that after a single p.o. administration of 60 mg of scutellarin to 20 healthy subjects, the *C*_max_ of scutellarin were low (87.0 ± 29.1 ng·mL^−1^). In this study, we found that SCU can influence the pharmacokinetics of RSV, despite low plasma concentrations *in vivo*. After co-feeding SCU, the AUC_0–24h_ and *C*_max_ of RSV in rats increased to 27.4% (*P* = 0.04) and 37.7% (*P* = 0.006), respectively. Moreover, SCU reduced the CL and CL/F of RSV by 29.2% (*P* = 0.009) and 28.1% (*P* = 0.008), indicating that SCU can have a considerable impact on the pharmacokinetic characteristics of RSV in rats (Fig. 3). In addition, Our previous intestinal absorption studies have shown that SCU does not affect the absorption rate constant or absorption coefficient of RSV (20). Therefore, this pharmacokinetic effect may not be attributed to the effect of SCU on the intestinal tract, instead, this inhibition on uptake may occur in the liver and the inhibitory effect of SCU on RSV may be mediated by liver OATP.

OATP transporters are classified into a large family of membrane proteins that mediate the sodium-independent cellular uptake of various amphipathic compounds, including hormones, bile acids, eicosanoids, environmental toxins, and many clinical drugs (24). Therefore, many drug interactions are mediated by OATP. In humans, both hOATP1B1 and hOATP1B3 genes are located on the short arm of chromosome 12 (gene locus 12p12), and they have up to 80% sequence homology. Both transporters have been described to be highly expressed in the human liver (25). OATP1B2 in the rodent is similar to hOATP1B1 and hOATP1B3, and these three transporters are uniquely expressed in the liver (26). OATP1B2 is most important for transporting unconjugated bile acids into the livers of mice (27). In addition, DeGorter *et al*. (28) found that OATP1B2 in mice is critical for the hepatic uptake of atorvastatin and rosuvastatin but not simvastatin acid. Mice OATP1B2 also plays an important role in the disposition of pravastatin and lovastatin (29). Higgins *et al*. (30) studied the pharmacokinetics of three statins (pravastatin, atorvastatin, and simvastatin) using OATP1B2-null mice, and also humanized OATP1B1 and OATP1B3 in mice. Therefore, OATP1B2 in rodents and hOATP1B1/1B3 are often studied in comparison.

According to a recent study, the targeted disruption of murine OATP1B2 may cause significant alteration to the disposition of prototypical drug substrates pravastatin and rifampin, and OATP1B2 has a high consistency with hOATP1B1 and hOATP1B3 in uptake function (18). In the present study, we propose that the changes in RSV pharmacokinetics are mainly attributed to the SCU mediated inhibition of OATP1B2 in rats. Due to the high degree of homology between human OATP1B1/1B3 and rOATP1B2, we believe that the studies on rats can provide a reference for studies on humans (31). However, the changes in OATPs expression between human and rat primary hepatocytes are not completely parallel, and there is no strict one-to-one mapping between human OATPs genes and rodent OATPs genes (32). In addition, it has been shown that single nucleotide polymorphisms of human OATP1B1/1B3 can affect the function of transporters, which may cause changes in the pharmacokinetics, efficacy, and side effects of certain drugs (33). Further human studies are needed to confirm our conclusions.

In summary, our study demonstrated for the first time that SCU has an inhibitory effect on hOATP1B3-mediated transport of RSV *in vitro*. These results suggest that SCU is the perpetrator of hepatic hOATP1B3 mediated DDI. Besides, it is speculated that SCU has a moderate inhibitory effect on the pharmacokinetics of RSV in humans mediated by hOATP1B3. This study is expected to show the clinical significance for patients taking SCU and hOATP1B3 substrate drugs simultaneously.

### Acknowledgements and Disclosures

This work was supported by China National Science Foundation for Distinguished Young Scholars (Grant No: 81202583); Jiangxi Education Department Science Plan (Key Project, Grant No: GJJ151335). The authors declare no competing interests.

## Data Availability

The raw data supporting the conclusions of this manuscript will be made available by the authors, without undue reservation, to any qualified researcher.

## Abbreviations

AUC: Area under the plasma concentration-time curve
*C*_max_: Peak plasma drug concentration
CL: Clearance
CL/F: Apparent clearance
DDI: Drug-drug interactions
GA: Glycyrrhizic acid
LC/MS/MS: Liquid chromatography-tandem mass spectrometry
OATP: Organic anion transporting polypeptide
RSV: Rosuvastatin
RIF: Rifampicin
SCU: Scutellarin
*t*_max_: Time to reach *C*_max_
*t*_1/2_: Elimination half-life
CMC-Na: Carboxymethylcellulose sodium

## References

[1] Yu WJ, Ma MY, Chen XM, Min JY, Li LR, Zheng YF, Li YS, Wang J, Wang Q (2017). Traditional Chinese medicine and constitutional medicine in China, Japan and Korea: a comparative study. Am J Chinese Med.

[2] Li PW, Qiang M (2018). Clinical benefits and pharmacology of scutellarin: a comprehensive review. Pharmacol Ther.

[3] Gao J, Chen G, He H, Liu C, Xiong X, Li J, Wang J (2017). Therapeutic effects of Breviscapine in cardiovascular diseases: a review. Front Pharmacol.

[4] Yang W, Li L, Xie YM, Zhuang Y, Yang W (2013). Treatment outcomes of parenterally administered dengzhan xixin for treatment of cerebral infaction based on real world hospital injection system data. Zhongguo Zhong Yao Za Zhi.

[5] Liu JM, Yang YX, Ye Y (2018). Effect of scutellarin on the plasma concentration and tissue distributions of rosuvastatin in rats. Chin J Clin Pharmacol.

[6] Lin SS, Tsai CL, Tu CY, Hsieh CL (2015). Reducing drug–herb interaction risk with a computerized reminder system. Ther Clin Risk Manag.

[7] Han BY, Yao YH (2014). Pharmacokinetic interaction and the mechanisms of combination of Chinese and western medicines. Medical Recapitulate.

[8] Mitchell RM, Hartmut J (2013). Metabolism and disposition of acetaminophen: recent advances in relation to hepatotoxicity and diagnosis. Pharm Res.

[9] Tornio A, Niemi M, Neuvonen PJ, Backman JT (2012). Drug interactions with oral antidiabetic agents: pharmacokinetic mechanisms and clinical implications. Trends Pharmacol.

[10] König J, Muller F, Fromm MF (2013). Transporters and drug-drug interactions: important determinants of drug disposition and effects. Pharmacol Rev.

[11] Gao C, Zhang H, Guo Z, You T, Chen X, Zhong D (2012). Mechanistic studies on the absorption and disposition of scutellarin in humans: selective OATP2B1-mediated hepatic uptake is a likely key determinant for its unique pharmacokinetic characteristics. Drug Metab Dispos.

[12] Alam K, Crowe A, Wang XY, Zhang PY, Ding K, Li L, Yue W (2018). Regulation of organic anion transporting polypeptides (OATP) 1B1- and OATP1B3-mediated transport: an updated review in the context of OATP-mediated drug-drug interactions. Int J Mol Sci.

[13] Liu JM, Guo Y, Liu KQ, Ye XY, Wang F, Xu Y, Xia CC (2020). Scutellarin inhibition of the rosuvastatin uptake in rat hepatocytes and the competition for organic anion transporting polypeptide 1B1 in HEK293T cells. Sci Rep.

[14] König J, Cui Y, Nies AT, Keppler D (2000). Localization and genomic organization of a new hepatocellular organic anion transporting polypeptide. J Biol Chem.

[15] Vavricka SR, Van MJ, Ha HR, Meier PJ, Fattinger K (2002). Interactions of rifamycin SV and rifampicin with organic anion uptake systems of human liver. Hepatology..

[16] Kitamura S, Maeda K, Wang Y, Sugiyama Y (2008). Involvement of multiple transporters in the hepatobiliary transport of rosuvastatin. Drug Metab Dispos.

[17] Cattori V, Hagenbuch B, Hagenbuch N, Stieger B, Ha R, Winterhalter KE, Meier PJ (2000). Identification of organic anion transporting polypeptide 4 (Oatp4) as a major full-length isoform of the liver-specific transporter-1 (rlst-1) in rat liver. FEBS Lett.

[18] Hani Z, Henriette E, Rommel G, Tirona, Melissa LC, Leslie AO, Nidhi A, Joe P, Jeffrey LS, Richard BK, Joseph AW (2008). Targeted disruption of murine organic anion-transporting polypeptide 1b2 (oatp1b2/Slco1b2) significantly alters disposition of prototypical drug substrates pravastatin and rifampin. Mol Pharm..

[19] Liu XN, Cheng J, Zhang GG, Ding WT, Duan LG, Yang J (2018). Engineering yeast for the production of breviscapine by genomic analysis and synthetic biology approaches. Nat Commun.

[20] Liu JM, Guo YM, Yang YX, Wang J, Wang F (2017). Influence of scutellarin on intestinal absorption of rosuvastatin in rats. Asia-pacific tradional medicine.

[21] Dong JJ, Olaleye OE, Jiang RR, Li J, Lu C, Du FF, Xu F, Yang JL, Wang FQ, Jia WW, Li C. Glycyrrhizin has a high likelihood to be a victim of drug–drug interactions mediated by hepatic organic anion-transporting polypeptide 1B1/1B3. Br J Pharmacol. 2018; 175:3486–503. 10.1111/bph.14393 PMC6086986 29908072

[22] You HS, Dong YL, Xing JF, Zhang CL, Wang MY (2007). Pharmacokinetic and tissue distribution study of scutellarin in rats. China Journal of Chinese Materia Medica.

[23] Chen XY, Cui L, Duan XT, Ma B, Zhong DF (2006). Pharmacokinetics and metabolism of the flavonoid scutellarin in humans after a single oral administration. Drug Metab Dispos.

[24] Stefan O (2019). Organic anion transporting polypeptide (OATP) transporter expression, localization and function in the human intestine. Pharmacol Ther.

[25] Ho RH, Tirona RG, Leake BF, Glaeser H, Lee W, Lemke CJ, Wang Y, Kim RB. Drug and bile acid transporters in rosuvastatin hepatic uptake: function, expression, and pharmacogenetics. Gastroenterology. 2006;130(6):1793–806. 10.1053/j.gastro.2006.02.034 16697742

[26] Pan Q, Zhang X, Zhang L, Cheng Y, Zhao N (2018). Solute carrier organic anion transporter family member 3A1 is a bile acid efflux transporter in cholestasis. Gastroenterology..

[27] Csanaky IL, Lu H, Zhang Y, Ogura K, Choudhuri S, Klaassen CD (2011). Organicanion-transporting polypeptide 1b2 (Oatp1b2) is important for the hepatic uptake of unconjugated bile acids: studies in Oatp1b2-null mice. Hepatology..

[28] DeGorter MK, Urquhart BL, Gradhand U, Tirona RG, Kim RB (2012). Disposition of atorvastatin, rosuvastatin, and simvastatin in oatp1b2−/−mice and intraindividual variability in human subjects. J Clin Pharmacol.

[29] Zaher H, Schwabedissen HE, Tirona RG, Cox ML, Obert LA (2008). Targeted disruption of murine organic anion-transporting polypeptide 1b2 (Oatp1b2/Slco1b2) significantly alters disposition of prototypical drug substrates pravastatin and rifampin. Mol Pharmacol.

[30] Higgins JW, Bao JQ, Ke AB, Manro JR, Fallon JK (2014). Utility of Oatp1a/1b-knockout and OATP1B1/3-humanized mice in the study of OATP-mediated pharmacokinetics and tissue distribution: case studies with pravastatin, atorvastatin,simvastatin, and carboxydichlorofluorescein. Drug Metab Dispos.

[31] Hagenbuch B, Meier PJ (2003). The superfamily of organic anion transporting polypeptides. Biochim Biophys Acta.

[32] Hagenbuch B, Meier PJ (2004). Organic anion transporting polypeptides of the OATP/SLC21 family: phylogenetic classification as OATP/SLCO superfamily, new nomenclature and molecular/functional properties. Pflug Arch.

[33] Gong IY, Kim RB (2013). Impact of genetic variation in OATP transporters to drug disposition and response. Drug Metab Pharmacokinet.

